# Responding to medetomidine in Philadelphia: narrative summary of the public health response to a new psychoactive substance

**DOI:** 10.1186/s13722-026-00713-y

**Published:** 2026-08-31

**Authors:** TaReva Warrick-Stone, Serge-Emile Simpson, Phil Durney, Dennis Goodstein, Catherine Abrams, Raymond Bobb, Kory London, Daniel Teixeira da Silva

**Affiliations:** 1 Department of Internal Medicine, Thomas Jefferson University, 1020 Walnut Street, Philadelphia, PA 19107 USA; 2 Jefferson Addiction Multidisciplinary Service, Thomas Jefferson University, 1020 Walnut Street, Philadelphia, PA 19107 USA; 3 Prevention Point Philadelphia, 2913 Kensington Avenue, Philadelphia, PA 19134 USA; 4 Division of Medical Toxicology, Jefferson Einstein Philadelphia, 5501 Old York Road, Philadelphia, PA 19141 USA; 5 Department of Pharmacy, Thomas Jefferson University, 1020 Walnut Street, Philadelphia, PA 19107 USA; 6 Substance Use Response, Guidance, and Education Program (SURGE), Health Federation of Philadelphia, 123 South Broad Street, Philadelphia, PA 19109 USA; 7 Merakey Parkside Recovery, 5000 Parkside Avenue, Philadelphia, PA 19131 USA; 8 Department of Emergency Medicine, Thomas Jefferson University, 1020 Walnut Street, Philadelphia, PA 19107 USA; 9 Substance Use Prevention and Harm Reduction (SUPHR), Philadelphia Department of Public Health , 123 South Broad Street, Philadelphia, PA 19109 USA

**Keywords:** Medetomidine, Adulterants, Withdrawal management, Substance use treatment, Public health, New psychoactive substance

## Abstract

**Background:**

Despite the emergence of medetomidine as the dominant adulterant in Philadelphia’s illicit opioid supply, coordinated public health responses are limited or non-existent in the literature. Thus, we summarize a recent Summit in Philadelphia of outpatient and inpatient service providers who specialize in the care of persons who use drugs to discuss medetomidine response readiness and building ongoing community readiness for potential destabilizing drug supply changes.

**Purpose:**

Describe a public health intervention that distributed information about practices across health care settings and mobilized key informants to identify and address barriers to treatment.

**Methods:**

Descriptive summary of the in-person Summit organized by the Division of Substance Use Prevention and Harm Reduction at the Philadelphia Department of Public Health and the Health Federation of Philadelphia’s Substance Use Response, Guidance, and Education program to review cases of medetomidine-associated withdrawal across outpatient, emergency department, hospital, and substance use treatment settings.

**Results:**

The authors reviewed the insights from attendees and grouped them into nine themes: overdose response, clinical recognition of withdrawal, monitoring withdrawal, treatment approach, patient care navigation, workforce development, testing and surveillance, mobile outreach, and reimbursement. The authors subsequently developed twelve summative recommendations to guide further public health interventions in Philadelphia and other communities where medetomidine may become a common adulterant in the drug supply.

**Conclusion:**

The insights gained regarding medetomidine withdrawal management will be shared with community organizations providing services to people who use drugs in Philadelphia and clarify specific barriers and areas for ongoing support. Other communities experiencing shifts in drug supply may benefit from this knowledge and similar information sharing colloquiums.

## Introduction

In 2024, medetomidine, an FDA-approved veterinary sedative, was detected in multiple opioid overdoses in Pennsylvania and Illinois [1–5]. These overdoses were characterized by bradycardia, hypotension, and prolonged sedation, with other regional hospitals reporting similar findings among patients presenting with an atypical opioid toxidrome [6]. Physiologically, medetomidine, an alpha-2 agonist, decreases the activity of norepinephrine release via hyperpolarization of norepinephrine-producing neurons in a negative feedback manner, resulting in sedation, analgesia, muscle relaxation, and anxiolysis [7]. 

Medetomidine had previously been detected sporadically alongside fentanyl and xylazine in illicit drug samples from Missouri, Colorado, Pennsylvania, California, and Maryland [1, 2, 4]. In fall 2024, frontline clinicians in Philadelphia’s hospitals and behavioral health settings reported severe and worsening withdrawal presentations attributed to medetomidine adulteration of the opioid supply. Supporting this clinical observation, between May and November 2024, the rate of medetomidine detected in Philadelphia’s fentanyl samples increased from 29% to 87%, with local emergency department (ED) presentations for severe opioid withdrawal more than doubling at the end of 2024 [8–10]. Recently, a Centers for Disease Control and Prevention (CDC) health advisory showed that while medetomidine is still concentrated in northeastern states, the geographic spread of medetomidine is expanding [11]. 

Experts in Philadelphia and Pittsburgh reported a novel and clinically distinct withdrawal syndrome attributed to medetomidine [12, 13]. Both reports described patients with typical signs of opioid withdrawal in addition to profound hypertension (blood pressures often exceeding 180/120 millimeters mercury [mmHg]) and tachycardia (heart rates above 140 beats per minute [bpm]), intractable vomiting, a unique tremor without clonus or hyper-reactivity, as well as incidence of posterior reversible encephalopathy syndrome (PRES) and myocardial injury. Ultimately, over 90% of the 175 patients in one report were admitted to intensive care units [14]. Subsequent case series reported relatively resistant withdrawal symptoms among patients receiving typical withdrawal management including opioid agonists, sedatives, ketamine and multiple alpha-2 agonist preparations [15, 16]. 

In response to a higher acuity withdrawal syndrome, experts in Philadelphia and Pittsburgh quickly adapted withdrawal protocols designed to target xylazine withdrawal to treat medetomidine-associated symptoms [15, 17, 18]. Many of these protocols involve high doses of alpha-2 agonists in combination with opioid agonists to stabilize severe symptoms and improve hemodynamics. Despite ongoing education and awareness initiatives, the stabilization of patients with medetomidine withdrawal remains a challenge due to the lack of familiarity with its presentation and an absence of accessible laboratory detection [19, 20]. 

Thus, the emergence of medetomidine in the drug supply necessitates addressing several critical public health needs: ensuring accurate recognition of this novel withdrawal syndrome across clinical settings, developing standardized treatment protocols adapted to medetomidine’s unique pharmacology, strengthening surveillance and drug checking infrastructure, and building workforce competency across both clinical and community-based services [21–23]. To disseminate emerging best practices and awareness, the Substance Use Prevention and Harm Reduction (SUPHR) Division of Philadelphia Department of Public Health (PDPH) coordinated efforts with the medical examiner’s office, surveillance drug checking, and syndromic surveillance by working closely with frontline clinicians to share early knowledge of the threat of medetomidine with the public through the Health Alert Network [24]. 

Here we describe findings from a citywide convening for organizations serving people who use drugs (PWUD) that was part of Philadelphia’s collaborative public health response to medetomidine. SUPHR, in collaboration with the Substance Use Response, Guidance and Education (SURGE) Program at the Health Federation of Philadelphia, hosted a case review Summit featuring inpatient and outpatient clinicians focused on strategic initial approaches to the management of medetomidine withdrawal. The intent was to provide a space for networking and knowledge sharing that would mobilize improvements at the individual, organizational, and systems level to better address medetomidine withdrawal and improve health outcomes for patients across multiple care settings. We examined the insights gained by attendees to propose updated best practices for mitigating the public health threat of medetomidine.

## Methods

Having previously developed recommendations for caring for individuals with xylazine-associated wounds in Philadelphia [25, 26] using a case review format adapted from the structure of Fetal and Infant Mortality Review [27], SUPHR and SURGE employed a similar approach for the Medetomidine Withdrawal Summit. The program was accredited for continuing medical education (CME), Pharmacy, Nursing and Social work credit through the American Academy of CME. Following opening remarks from the SUPHR director were case presentations, a panel discussion with case presenters to address questions from attendees, small group breakout sessions, large group debrief, then closing remarks.

In total, 115 people attended the in-person event on November 6, 2025. Most were health professionals: 44 in medical settings and 45 in behavioral health. Attendees included physicians, registered and advanced practice nurses, pharmacists, social workers, substance use navigators, certified recovery specialists, and harm reduction outreach workers from primary care, mobile outreach, and hospital-based ED or inpatient medicine across the three local academic health systems, correctional health, federally qualified health centers, crisis response centers, residential drug treatment facilities, and opioid treatment programs. Additionally, 25 public health and City of Philadelphia employees and seven academic researchers attended. Several participants from outside the city traveled from Pittsburgh, New Jersey, and New York.

### Summary of case presentations

Four vignettes, based on real individual cases, were presented to attendees. These cases were modified to emphasize critical points of care in the era of medetomidine withdrawal, and to fully de-identify the encounters.

#### Case 1 - Ambulatory care

The first case focused on a young male with active injection drug use, housing instability, and untreated hepatitis C, who had recently arrived at a large, shared-room supportive housing facility that has an attached walk-in primary care and urgent care clinic. The proximity of housing and medical services allowed for walk-in visits and the opportunity for staff engagement. Over several encounters, the clinical team assessed the patients’ goals for opioid use treatment, obtained phlebotomy to screen for HIV, viral hepatitis, diabetes, thyroid, and renal disease, and prescribed medications for HIV prevention and hepatitis C treatment. The patient visited several times to interact with case management and medical staff, thus offering multiple opportunities to check vital signs and adjust clonidine dosing when his drug use decreased and his blood pressure increased to 164/91 mmHg. Staff were prepared to support this patient at future encounters, as oral and intramuscular (IM) medications for withdrawal management were available on site, and the withdrawal protocol provided staff with clear indications and parameters for use. The clinical team also had a protocol for escalating care to the emergency department via 911 if necessary for: vomiting, seizures, BP > 185/100 mmHg, abnormal neurologic exam, or Clinical Opiate Withdrawal Scale (COWS) score > 20. This case demonstrated how low barrier, patient-centered, and co-located care in residential facilities can be a very effective model in an era of ever more toxic street drugs.

#### Case 2 - Inpatient hospitalization

The second case described the longitudinal hospital course of a young male with opioid use disorder (OUD) and housing instability who required admission after presenting to the ED with withdrawal. Although the patient initially appeared to have mild withdrawal with last use of injection drugs a few hours prior to presentation, his symptoms rapidly escalated to severe withdrawal within four to six hours despite aggressive oral pharmacologic management. The patient developed hallmark features of severe medetomidine withdrawal, including tachycardia (heart rate 140 beats per minute), hypertension (182/95 mmHg), intractable nausea and vomiting, and encephalopathy with hypoactivity. This constellation of symptoms limited the safety of administering oral alpha-2 agonist therapy like clonidine. Approximately twelve hours after presentation, the patient required escalation to the intensive care unit (ICU) for a high-dose intravenous (IV) dexmedetomidine infusion. Over the subsequent 48 h, the infusion was successfully weaned through an aggressive cross-taper to oral and transdermal alpha-2 agonists, including clonidine, tizanidine, and guanfacine. Concomitantly, the patient completed a low-dose buprenorphine induction with transition to long-acting injectable buprenorphine prior to discharge. The patient was linked to a low barrier outpatient clinic for ongoing management of clonidine taper, medications for opioid use disorder (MOUD), and primary care. This case highlighted the rapid progression of medetomidine withdrawal and the narrow therapeutic window for preemptive oral pharmacologic management.

#### Case 3 - Residential rehabilitation treatment program

The third case highlighted the challenges of managing the rapidly worsening withdrawal syndrome associated with opioids and medetomidine within an inpatient substance treatment setting. The facility provided American Society of Addiction Medicine (ASAM) 3.5 and 3.7 levels of care focusing on withdrawal management, with a limited number of oral and IM medications on formulary in addition to ongoing behavioral support [28]. The team noted that level 3.5 and 3.7 facilities are not required to have a medical provider on site 24 hours per day. Clinical staff to patient ratios may also be insufficient for managing urgent or escalating needs, without referral to a higher level of care (hospital). The patient was a young male with housing instability, a history of both hepatitis C and B, and active injection drug use who presented as an unscheduled “walk-in” admission for residential treatment. On admission, his blood pressure (174/98 mmHg) and heart rate (108 bpm) were elevated, but he was calm. Approximately four to five hours later he became restless and while no longer tachycardic, his blood pressure had increased to 203/108 mmHg. He was given a variety of oral medications to treat his symptoms, but started to vomit, which did not resolve with additional oral or IM medications. Subsequently, staff noted a change in his mental status and described him as awake, but unable to respond to questions. Thus, 911 was called and he was taken to the hospital. This case illustrated the pressing need for updated treatment protocols and early intervention for anticipated medetomidine withdrawal in non-hospital care settings.

#### Case 4 - Emergency department

The final case detailed the characteristics of medetomidine overdose and withdrawal through the clinical course of a young female patient who received naloxone by both a bystander and emergency medical services (EMS) personnel prior to arrival in the ED. Her initial vitals included low blood pressure (96/42 mmHg) and bradycardia (heart rate 46 bpm) attributed to the unintentional overdose. Within two hours, her hemodynamics shifted rapidly to severe hypertension (192/108 mmHg), tachycardia (heart rate 134 bpm) with symptoms of intractable vomiting and myalgias. Initial stabilization included a variety of IV, IM, orally disintegrating, and transdermal (TD) medications including hydromorphone, ketamine, olanzapine, diphenhydramine, clonidine and scopolamine. The goal of this regimen was to administer oral clonidine (0.6 mg x three) to control her withdrawal. After a few hours, she began to improve and did not require a dexmedetomidine infusion or ICU level of care but was admitted to the hospital for ongoing withdrawal management, started on methadone maintenance, and discharged with follow-up scheduled at a methadone clinic, which was her preference. This case underscored the importance of vital sign monitoring to track the progression of withdrawal as well as the importance of early and aggressive management to control symptoms in the critical window of acuity.

### Summary of panel discussion

Following the cases, the presenters answered several questions as a panel, facilitated by a moderator. The presenters included emergency medicine physicians, hospital-based addiction medicine and toxicology consultants, an ambulatory addiction and primary care physician, a behavioral health and opioid treatment-based physician, and a clinical pharmacist experienced in managing acute withdrawal syndromes. Prior to the Summit, the registration survey solicited questions from the attendees. The session emphasized the lack of standard treatment protocol and, like all withdrawal management, tailoring treatment to an individual’s needs, with particular attention to the severity of past withdrawal, such as history of admission to an ICU for management of medetomidine withdrawal, as a better indicator of future withdrawal than current volume of drug use [29]. The panelists agreed that the COWS score [30] appeared to underestimate the severity of withdrawal in patients afflicted by a hypoactive delirium, which many recognized as a distinctive feature of severe medetomidine withdrawal. The COWS score remained useful nonetheless in tracking response to therapy and improvement of withdrawal in many cases. The panel acknowledged that there is currently no validated instrument for measuring alpha-2 withdrawal and attendees were encouraged to use elevated heart rate and blood pressure as more reliable indicators of medetomidine withdrawal severity and to titrate withdrawal management such as dexmedetomidine infusions to vital signs rather than mental status.

When asked about managing withdrawal outside the hospital, the panelists emphasized the need for patients to tolerate oral medications. If patients are vomiting or having difficulty interacting or following instructions, they generally need to be transferred to a higher level of care. However, if staff can provide parenteral, sublingual, or TD medications coupled with frequent reassessments of mental status, heart rate, and blood pressure, transferring patients with milder symptoms may not be necessary. Panelists recommended outpatient providers to invest and train staff in the use of sphygmomanometer blood pressure monitors and pulse oximeters so that they may identify both overdose and withdrawal syndromes. Panelists identified that patients who become “quiet” most need intervention, emphasizing the need to monitor for decreased responsiveness and transfer to a higher level of care when there is a change in mental status.

Clonidine was discussed at length as the mainstay of withdrawal management. There are three doses available of both the oral medication (0.1 milligram [mg], 0.2 mg, 0.3 mg), as well as the TD patch (0.1 mg/24 hour [hr], 0.2 mg/24 hr, 0.3 mg/24 hr). While usually available in the hospital, clonidine TD patches may not be covered by insurance in the outpatient setting despite TD clonidine currently being on Pennsylvania’s medical assistance preferred drug list [31]. Panelists advised that it takes 24–48 hours for the TD clonidine patch to offer any withdrawal relief [32], and they mentioned that oral clonidine tablets can be absorbed sublingually with no loss of efficacy when a patient is unable to swallow pills [33]. While the outpatient expert on the panel had clonidine on-site, it was unclear whether that practice was common among ambulatory care settings.

Concurrent opioid withdrawal management is necessary in the setting of medetomidine withdrawal, and both IV and oral hydromorphone and methadone were identified as useful opioid replacement agents that can be titrated to effect. Although gabapentin does not directly treat the pathophysiologic cause of medetomidine withdrawal, panelists identified it as a helpful adjunctive therapy for managing mild to moderate medetomidine withdrawal symptoms. However, they noted that patients can become physiologically dependent upon high-dose gabapentin.

Managing medetomidine withdrawal in pregnant patients was discussed as particularly challenging. Antiemetics typically used to control nausea in pregnant patients were believed to be ineffective for patients in withdrawal, and although antihypertensives used to control elevated blood pressures in pregnancy may eventually have a dose dependent effect, they do not treat the underlying mechanism of withdrawal in the acute phase [34]. Panelists agreed that the benefits of using agents like clonidine in pregnancy outweighed the risks of undertreated medetomidine withdrawal (see Table 1) [35]. If benzodiazepine therapy is required during pregnancy in the setting of acute withdrawal, lorazepam is the preferred agent due to its lack of active metabolites and shorter half-life.

**Table 1:** Recommended alpha-2 agonists for treating medetomidine withdrawal

Alpha-2 Agonist	Routes of Administration	Dosing for Medetomidine Withdrawal Management	Notes/Clinical Considerations	Pregnancy Considerations
Dexmedetomidine	IV, sublingual, intranasal (off-label)	0.2–2.5 mcg/kg/hr (titration to vital signs)	Hemodynamic effects; restrictions on level of care for monitoring in hospital use	Limited human data; No teratogenicity shown in animal models; Benefits of use in severe withdrawal likely outweighs risks, use with caution
Clonidine	TD, PO	PO: 0.1-0.6 mg, three times daily; TD:0.3 mg/24hr x one to three patches weekly	Hemodynamic effects	Most human data; No evidence of congenital defects; May use with routine monitoring
Tizanidine	PO	2 mg-6 mg, three times daily	QTc prolongation; Limited hemodynamic effects; Renal and hepatic dosing considerations	Limited human data; Animal data suggests toxicity, but not teratogenicity; Avoid when possible
Guanfacine	PO	0.5-3 mg, three times daily	Fewer hemodynamic effects	Limited human data; No evidence of congenital defects from available data; May use with routine monitoring

PO = oral, TD = transdermal, mcg = microgram, kg = kilogram, hr = hour, QTc = corrected QT interval

There were four 30-minute breakout sessions structured to allow smaller group discussions regarding the following topics: [1] key takeaways from the Summit [2], actionable changes given new knowledge [3], barriers to implementing change, and [4] outside support that would improve the quality of care delivered to people impacted by medetomidine. The small group sessions were neither recorded nor transcribed verbatim. Facilitators for each group took notes, which the authors later compiled, categorized into themes based on similarities, and reviewed. We modified original phrasing of key takeaways as little as possible to provide clarity and sought citations for evidence-based statements.

## Results

Attendees reported gaining new knowledge related to responding to overdose, the clinical recognition of medetomidine withdrawal, monitoring withdrawal, and the approach to treatment. Additionally, participants identified the need for, and barriers to, action, especially regarding helping patients navigate care between settings, workforce development and training, testing and surveillance of medetomidine, mobile outreach, and reimbursement for care. See Table 2 for examples of representative key takeaways. Overall, 74 insights across nine themed categories were identified: overdose response, clinical recognition of withdrawal, monitoring withdrawal, treatment approach, patient care navigation, workforce development, testing and surveillance, mobile outreach, reimbursement.

**Table 2:** Medetomidine summit breakout session takeaways

Overdose Response	• Despite its apparent inability to reverse the sedation and vital sign changes caused by medetomidine in overdose, naloxone remains a lifesaving medication that can reverse the potentially lethal respiratory depression caused by fentanyl. • Overdose assessment in the setting of medetomidine adulteration is most effective when focused more on respiratory status and less on the level of consciousness. • Providers are adjusting how they interpret responsiveness versus breathing following reversal. • The rise in the number of patients transported to EDs after overdose reversal is likely driven by persistent bradycardia and hypotension lasting hours after overdose despite multiple doses of naloxone.
Clinical Recognition of Withdrawal	• When people experience medetomidine withdrawal, they may not be able to answer questions or communicate with caregivers. This hypoactive delirium is a new and under-recognized phenomenon for many providers. It is reflective of critical illness, not patient apathy. • Individuals who appear “easier” to manage, with underestimated COWS scores due to hypoactive delirium, may be experiencing the worst withdrawal. • The COWS score may underestimate the severity of withdrawal if a patient is suffering from hypoactive delirium. This generated uncertainty regarding the utility of a COWS score in determining medetomidine withdrawal severity. • To an unfamiliar practitioner, the delirium of severe medetomidine withdrawal can be confused with the profound sedation of intoxication. • A patient’s history of recent withdrawal features, including time to symptom onset, symptom severity, and prior ICU admissions, can often predict the course of subsequent withdrawal events. • Recognizing the presence of simultaneous alpha-2 agonist withdrawal and opioid withdrawal syndromes will allow for more effective management of both. • It is challenging to determine when a patient requires ED evaluation, but vomiting has emerged as a consistent indicator for the need to escalate care. • Individuals with intractable nausea and vomiting from withdrawal may at times present with bradycardia due to increased vagal tone, which can confound attempts to distinguish withdrawal from intoxication using heart rate alone.
Monitoring Withdrawal	• Familiarity with how to identify worsening withdrawal is key knowledge for all direct service providers including non-clinical staff, peer navigators, first responders, as well as clinicians. • Hypoactivity can be an early indicator of medetomidine withdrawal. • Sphygmomanometers and pulse oximeters are tools for monitoring blood pressure and heart rate to determine the severity and progression of medetomidine withdrawal in both hospital and non-hospital settings. • Adequate staffing is necessary for frequent vital sign checks. • The COWS score may be helpful in tracking response to withdrawal treatment.
Treatment Approach	• The rapidly increasing toxicity of street opioids has forced a significant shift in withdrawal management, utilizing medications previously reserved for the ICU to control critical illness. The increased volume of patients seeking withdrawal treatment has strained the entire health system, affecting both behavioral and medical services. • Effective and available medications vary by institution and care setting. • Clonidine is a helpful medication for control of medetomidine withdrawal. It is generally safe and well-tolerated at dose units between 5–15 mcg/kg. Dosing can be titrated according to symptom severity. • Control of symptoms is time sensitive—there is often a narrow window of opportunity to give patients oral medications early during acute withdrawal. • Clonidine doses for medetomidine withdrawal management (i.e., 0.3 mg three to four times daily), are substantially higher than doses for chronic hypertension management, and are the first line intervention for effectively treating medetomidine withdrawal. • Sublingual formulations of dexmedetomidine and IV forms of clonidine exist, which may be an additional agent for managing medetomidine withdrawal, but have not yet been widely used due to cost. • Patients with severe medetomidine withdrawal who cannot tolerate oral alpha-2 agonists may require an IV dexmedetomidine infusion and can start clonidine as soon as possible to facilitate titration off dexmedetomidine infusion. • Using multiple alpha-2 agonists and multiple routes of administration is not standard practice (e.g., PO and TD clonidine; using clonidine and tizanidine), however, may be necessary to adequately control withdrawal symptoms. • Chlorpromazine (first-generation antipsychotic) and similar anti-dopaminergic medications may help with symptoms such as “brain zaps,” as well as severe vomiting. • Multiple agents have been effective in controlling nausea and vomiting well enough to preserve the ability to give oral clonidine, so choose whichever is available in the setting of formulary restrictions (e.g., IM haloperidol off label if no other antiemetic available in behavioral health setting can be administered). • Treatment of underlying OUD by offering patients the opportunity to start MOUD can reduce exposure to medetomidine in the fentanyl supply. · Long-acting injectable buprenorphine (LAIB) offers some protection against respiratory arrest in overdose, even if patients return to drug use. · Providers can utilize weekly LAIB as a quick start and transition to monthly LAIB on subsequent doses. · LAIB will not treat or prevent medetomidine withdrawal, so patients may need education regarding which medications are effective for treating medetomidine withdrawal symptoms.
Patient Care Navigation	• Criminalization of drug use complicates patient care and engagement. • Many patients face housing insecurity, which can limit follow-up and continuity of care. • Patients are not always ready for inpatient (hospital) care when assessed outside the hospital. This may be due to negative experiences from stigmatized care provided at their local hospitals; so many may choose to delay care or present to residential substance treatment “rehab” or medically monitored withdrawal “detox” centers. However, these facilities may not have the capacity to manage the acuity of care when patients become ill from withdrawal, resulting in the transfer of critically ill patients from “detox” centers to hospitals. Effectively, there are few options for people to stop using without getting sick. • Lack of permanent addresses complicates contact and care coordination. • Many patients lack cell phones, making communication difficult. • The severity of medetomidine-associated withdrawal may make it especially difficult for people to enter and sustain recovery. • Emergency department wait times can be intolerable for patients in withdrawal. Thus, patients are reluctant to go to hospitals because of unpredictable or long wait times to receive care. • Better documentation is needed to communicate to patients why some patients are admitted versus discharged from the emergency department. • Lowering hospital admission thresholds would offer a higher level of care to patients who are at risk of severe withdrawal. • Hospitals are concerned that they are not licensed as medically managed withdrawal “detox” facilities and are therefore unclear about who can and cannot be admitted. • Patients not yet in active withdrawal have difficulty accessing hospital-based care. • ICU beds are a limited resource under strain because of severe medetomidine withdrawal when symptoms require the use of dexmedetomidine infusion. • Preventative care, such as permanent supportive housing, could reduce the use of emergency departments and inpatient hospital services and financial strain on the system [36].
Workforce Development	• Stigma around substance use disorder remains a major barrier. • Free, accredited training across disciplines will continue to promote system improvement. • Sharing success stories shifts perception and reduces stigma. • Staff who can recognize withdrawal, estimate its severity, and assess its progression help keep patients safe. • Night staff in residential substance treatment settings often do not care for acutely ill individuals and may need additional training to reach the same competency as day staff who more regularly assess patients in withdrawal and provide medications to stabilize acute or worsening symptoms. • Emphasizing trauma-informed care in training can remind staff that “poor behavior” among patients who use drugs is rarely malicious. • Nursing care can significantly influence whether someone has a positive or negative treatment experience. • Staff may require training to recognize hypoactive delirium and the risks associated with misinterpreting this sign of medetomidine withdrawal as volitional apathy. • Navigator and certified recovery specialist (CRS) roles could be expanded to include meeting with patients before discharge for aftercare planning. • CRS staffing is unstable due to funding gaps, leading to job losses and hiring challenges. • Overnight CRS and social worker coverage remain limited.
Testing and Surveillance	• The drug supply is extremely complicated and rapidly changing. • The drug supply evolves faster than surveillance systems can adapt. • Providers are often a year, or more, behind shifts in the drug supply. • The cost of testing for medetomidine and its metabolites remains a major barrier to broader use in hospitals.(20) • Medetomidine testing strips are most effective when used in mobile and community healthcare settings. • The accuracy of medetomidine test strips for checking drug samples for the adulterant relies on proper sample preparation but were assessed to have 100% sensitivity in a preliminary data set [37]. • The value of testing with medetomidine strips is questionable when the prevalence of medetomidine in the local drug supply is known to be extremely high from more reliable surveillance efforts.
Mobile Outreach	• Mobile providers may lack clarity on how to manage patients who refuse hospitalization. • There is uncertainty around whether it is safe to continue clonidine for patients who want to treat medetomidine withdrawal but are not interested in starting MOUD to reduce the use of fentanyl that further exposes them to medetomidine. • Street teams benefit from having medications stocked and ready to dispense rather than relying on prescriptions. • Street teams have expanded the list of medications they want to carry, with clonidine highlighted as particularly valuable. • Mobile providers need better knowledge of in-hospital and ICU care practices to set expectations for patients. • Stronger relationships between physicians and mobile outreach teams would improve post-discharge follow-up.
Reimbursement	• Federal harm-reduction funding is limited, affecting state-level efforts. • Reimbursement for medically monitored withdrawal “detox” and residential substance treatment “rehab” is “abysmal.” • Medically monitored withdrawal “detox” reimbursement is approximately one-tenth of hospitalization rates. • Providers struggle with the decision between hospital admission and “detox” due to financial constraints. • International Classification of Diseases (ICD) codes for medetomidine withdrawal are needed to aid with epidemiologic tracking and hospital reimbursement. • Poor understanding of coding limits revenue generation and system sustainability.

ED = emergency department; COWS = clinical opiate withdrawal scale; ICU = intensive care unit; mcg = microgram; kg = kilogram; mg = milligram; IV = intravenous; PO = oral; TD = transdermal; IM = intramuscular; OUD = opioid use disorder; MOUD = medications for opioid use disorder; LAIB = long-acting injectable buprenorphine; CRS = certified recovery specialist; ICD = international classification of diseases

## Discussion

This narrative description of a public health intervention designed to distribute information about managing medetomidine withdrawal across health care settings highlights the important role of health departments in supporting rapid adaptation among health systems in response to a new psychoactive substance (NPS) such as medetomidine. Changes in the local drug supply have burdened health systems and impacted how patients can access and sustain engagement in care [14]. Crucially, information exchange systems can communicate accurate, reliable information about NPS to PWUD, health professionals, other care providers, and the public.

With the emergence of xylazine, one study of healthcare providers’ knowledge and perceptions found a need for improved data sources so that providers were not relying on delayed or biased data to inform the care of their patients [38]. Thus, Philadelphia’s response to medetomidine emphasized confidently attributing the recent harms of naloxone-resistant overdose and life-threatening withdrawal to medetomidine exposure [20, 39]. While challenges exist in attributing adverse outcomes to a specific NPS given the high incidence of polysubstance use, forensic toxicology’s analytical approaches can detect, identify, and quantify NPS within drug samples or human biological samples [40, 41]. 

For example, the Center for Forensic Science Research and Education (CFSRE)’s NPS Discovery, an early warning system in the United States, monitors NPS exposures throughout forensic casework [42]. The SUPHR Division of PDPH started collaborating with CSFRE’s NPS Discovery in September 2020 to accurately assess the drug supply in Philadelphia for xylazine adulteration. Importantly, this collaboration includes PA Groundhogs, an organization that works directly with PWUD to implement drug checking and harm reduction strategies that rapidly communicate critical information obtained through empiric observation in an actionable and beneficial way [43]. Surveillance can also occur at the individual level through the recent development of rapid medetomidine testing strips [44]. 

Although health and community workers are a crucial point of contact for drug-related issues, information, and advice, workforce research has demonstrated the little existing support and education available for these workers to discuss NPS or shifts in the drug supply [45]. As such, drug alerts that target health and community workers can facilitate broader dissemination of up-to-date information and prepare services to respond to drug market fluctuations or unusual drug-related events [45]. Currently, Health Alerts and CHART publications created by SUPHR communicate changes in the Philadelphia drug supply. SUPHR’s first publication related to medetomidine on May 13, 2024 was a Health Alert: “In Philadelphia, medetomidine, a potent non-opioid veterinary sedative, has been detected in the illicit drug supply” that described medetomidine, symptoms of medetomidine exposure, and suggested clonidine as an appropriate therapy for withdrawal [6]. Medetomidine became the primary topic of discussion for the quarterly hospital workgroup hosted by SUPHR from October 2024 – October 2025, as well as its monthly meetings with hospital addiction medicine consult teams.

SUPHR subsequently published a more detailed Health Alert in December 2024: “Hospitals and behavioral health providers are reporting severe and worsening presentations of withdrawal among PWUD in Philadelphia.”[9] Additional publications related to medetomidine were released by SUPHR in May 2025, “CHART: Changes in Philadelphia’s Drug Supply and Substance-Use Related Emergency Department Visits”[10] and a June 2025 Health Update: “Responding to overdose and withdrawal involving medetomidine” that offered more detailed guidance for managing overdose or withdrawal related to medetomidine and additional information for non-hospital settings such as when to consider transfer to the hospital [19]. Health alerts offer a timely, lower-barrier method for disseminating targeted and relevant information in a way that supports workforce capacity in responding to adverse events.

However, many care settings may need additional support to shift clinical protocols and prepare the workforce to provide high quality care. As much of our understanding of NPS comes from case reports and case series [40], Philadelphia’s Medetomidine Withdrawal Summit in November 2025 allowed for in-person instruction regarding early detection of withdrawal, emerging clinical management, and general networking to improve individual, organizational, and system level responses to medetomidine exposure. The cases presented by experienced providers described interventions in the outpatient, ED, hospital, and substance use treatment settings. Education emphasized identifying the signs and symptoms unique to medetomidine-exposed patients, characterizing the strategies and tools for treating and monitoring symptoms that indicate the progression of withdrawal, as well as recognizing when escalation or transfer of care might be necessary. A panel of experts facilitated further discussion. Finally, attendees representing organizations across the city’s providers of services for PWUD met in smaller breakout sessions to discuss new knowledge, ideas for change, and barriers to improved quality of care.

From these, we generated twelve recommendations:

- Policy and funding for community-based drug checking programs that provide rapid and accurate results on the local drug supply is necessary for mobilizing a public health response to the emergence of medetomidine;
- Existing networks of providers who support harm reduction for PWUD must be included in disseminated information about medetomidine and prevention of unwanted medetomidine exposure;
- Naloxone remains the first line to reverse opioid overdose. If concerned about medetomidine intoxication, assessment for redosing should be evaluated only if the patient is not breathing adequately as patients may remain sedated;
- Close monitoring for rapid shifts between the withdrawal and intoxication phase of medetomidine is required to prevent severe medical complications of withdrawal;
- Heart rate trends can help distinguish between the intoxication and withdrawal phase of medetomidine: bradycardia correlates with intoxication, while extreme tachycardia signifies withdrawal;
- Due to the risk of neurologic and cardiac injury, people with profound hypertension with or without tachycardia should be referred for an acute medical evaluation until a system to more closely monitor patients at risk of decompensation in community sites can be operationalized;
- When oral medications can be tolerated, clonidine tablets are the mainstay of withdrawal management, and can be administered sublingually with no loss of efficacy;
- Anecdotally, dopamine antagonist agents (such as metoclopramide, olanzapine, prochlorperazine, haloperidol, and chlorpromazine) more effectively treat the nausea and vomiting associated with medetomidine withdrawal to allow the use of oral clonidine;
- Prior need for dexmedetomidine infusion as part of initial management of severe medetomidine withdrawal is a better predictor of need for similar management than currently reported volume of use;
- Sustainability of both the medical and behavioral health systems requires that reimbursements reflect the complexity of care being provided to manage medetomidine-associated withdrawal; and.
- Given the downstream implications for epidemiologic surveillance and healthcare reimbursement, an ICD code for medetomidine withdrawal is needed.
- Funding free accredited education initiatives for all members of the medical and behavioral care team is necessary to provide high quality care and prevent harms to people exposed to medetomidine.

There are limitations to this study given its descriptive nature. Future studies could include quantitative surveys and qualitative evaluations of attendee confidence in recognizing, treating, and counseling patients exposed to medetomidine and whether participation in the Medetomidine Withdrawal Summit changed confidence. Future efforts to build knowledge around medetomidine should continue to be collaborative with opportunities for information exchange by a wide range of providers and community members. For instance, people with lived experience of medetomidine exposure should be incorporated into, and compensated for, medical training of providers who serve PWUD. Transitions from acute care settings to outpatient management, including discharge planning, medication coordination, and ongoing tapering support should be a future focus for ongoing educational endeavors. Critical gaps remain in outpatient tapering protocols, maintenance of cravings for alpha-2 agonists, as well as coordination with primary care and other addiction treatment providers.

## Conclusion

Proactive coordination among hospitals, health care organizations, and public health agencies to share observations, data, and expertise is essential to respond to the emergence of new psychoactive substances in the drug supply such as medetomidine. Case-based education regarding medetomidine withdrawal and emerging clinical management in different healthcare settings, combined with in-person networking was effective in generating individual, organizational, and system level interventions for improved quality of care.

## Data Availability

No datasets were generated or analysed during the current study.

## Abbreviations

ED: Emergency department
CDC: Centers for Disease Control and Prevention
mmHg: Millimeters mercury
bpm: Beats per minute
PRES: Posterior reversible encephalopathy syndrome
SUPHR: Substance Use Prevention and Harm Reduction
PDPH: Philadelphia Department of Public Health
PWUD: People who use drugs
SURGE: Substance Use Response
IM: Intramuscular
COWS: Clinical Opiate Withdrawal Scale
OUD: Opioid use disorder
ICU: Intensive care unit
IV: Intravenous
MOUD: Medications for opioid use disorder
ASAM: American Society of Addiction Medicine
EMS: Emergency medical services
TD: Transdermal
mg: Milligram
hr: Hour
PO: Oral
mcg: Microgram
kg: Kilogram
QTc: Corrected QT interval
LAIB: Long-acting injectable buprenorphine
CRS: Certified recovery specialist
ICD: International classification of diseases
NPS: New psychoactive substance
CFSRE: Center for Forensic Science Research and Education
